# Vaccine Preventability of Meningococcal Clone, Greater Aachen Region, Germany

**DOI:** 10.3201/eid1603.091102

**Published:** 2010-03

**Authors:** Johannes Elias, Leo M. Schouls, Ingrid van de Pol, Wendy C. Keijzers, Diana R. Martin, Anne Glennie, Philipp Oster, Matthias Frosch, Ulrich Vogel, Arie van der Ende

**Affiliations:** University of Wuerzburg, Wuerzburg, Germany (J. Elias, M. Frosch, U. Vogel); National Institute for Public Health and Environment (RIVN), Bilthoven, the Netherlands (L.M. Schouls, I. van de Pol); Academic Medical Center, Amsterdam, the Netherlands (W.C. Keijzers, A. van der Ende); Institute of Environmental Science and Research, Porirua, New Zealand (D.R. Martin, A. Glennie); Novartis Vaccines, Siena, Italy (P. Oster); 1 These authors contributed equally to this article.

**Keywords:** Meningococcal infections, meningococcal vaccine, bacterial typing techniques, outbreak, bacteria, Germany, the Netherlands, Greater Aachen Region, research

## Abstract

Emergence of serogroup B meningococci of clonal complex sequence type (ST) 41/44 can cause high levels of disease, as exemplified by a recent epidemic in New Zealand. Multiplication of annual incidence rates (3.1 cases/100,000 population) of meningococcal disease in a defined German region, the city of Aachen and 3 neighboring countries (Greater Aachen) prompted us to investigate and determine the source and nature of this outbreak. Using molecular typing and geographic mapping, we analyzed 1,143 strains belonging to ST41/44 complex, isolated from persons with invasive meningococcal disease over 6 years (2001–2006) from 2 German federal states (total population 26 million) and the Netherlands. A spatially slowly moving clone with multiple-locus variable-number tandem repeat analysis type 19, ST42, and antigenic profile B:P1.7–2,4:F1–5 was responsible for the outbreak. Bactericidal activity in serum samples from the New Zealand MeNZB vaccination campaign confirmed vaccine preventability. Because this globally distributed epidemic strain spreads slowly, vaccination efforts could possibly eliminate meningococcal disease in this area.

## Abstract 2

An emerging serogroup B clone can be prevented by vaccines.

Our work describes the epidemiology of invasive meningococcal disease (IMD) caused by meningococci of clonal complex (cc) 41/44 in the Netherlands and the 2 bordering German states Lower Saxony and North-Rhine-Westphalia during 2001–2006. *Neisseria meningitis* is a gram-negative bacterium that occasionally causes invasive disease in humans, primarily meningitis or sepsis (*1*). Notwithstanding low incidence rates in most industrialized countries, IMD remains a serious public health problem because of its predilection for affecting young persons and its ≈10% death rate despite antimicrobial drug treatment. In contrast to the meningitis belt in Africa, where epidemic waves cause incidence rates up to 300 cases/100,000 population (*2*), epidemics or case clusters are rare in industrialized countries (*3*). Meningococci are antigenically diverse bacteria that can be divided into 12 serogroups by variation of their polysaccharide capsules. Despite increased findings of serogroup C meningococci in several countries, serogroup B has clearly controlled the epidemiology of IMD in western Europe for the past 20 years. Dominance of serogroup B has further been compounded by numerous vaccination campaigns with polysaccharide C conjugate vaccine leading to a decline in serogroup C disease (*4*). Unfortunately, the serogroup B polysaccharide is an unsuitable vaccine antigen because of poor immunogenicity. Despite substantial progress in the development of vaccines based on membrane-associated antigens (*5**,**6*), a universal vaccine against meningococci has yet to be licensed.

Typing of *N. meningitidis* is critical for tracking transmissions and recognition of disease clusters. In recent years, focus has shifted to portable molecular typing methods with high discriminatory power. The preferred method for sequence-based typing of meningococci is multilocus sequence typing (MLST) (*7*), which enables identification of strains belonging to hypervirulent clonal complexes responsible for most cases of the invasive disease (*8*). MLST is complemented by antigen sequence typing of the variable regions of the outer membrane proteins PorA and FetA (*9*). Moreover, multiple-locus variable-number tandem repeat analysis (MLVA), which shows slightly higher discriminatory ability than MLST (*10*), represents a recent addition to the arsenal of portable typing methods for *N. meningitidis*.

Differences in the antigenic makeup of meningococcal clonal complexes (cc) (*11*) likely influence reported disparities in spatiotemporal spread. Whereas strains belonging to the multilocus sequence type (ST) 5 complex (cc5/subgroup III) (*12*) and ST-11 complex (cc11/ET-37 complex) (*13*) depend on migration to survive, strains of the ST41/44 complex (cc41/44/lineage 3) have been described as causing stationary and persistent hyperendemic disease, as exemplified by the New Zealand serogroup B epidemic, which lasted more than a decade (*14*).

cc41/44 is a large hypervirulent complex that revolves around 2 STs instead of 1 central ST, namely ST41 and ST-44 (*15*). It was first described in the Netherlands in the 1980s (*16*), where it caused a substantial increase in disease incidence (*17**,**18*). Subsequently, this lineage was reported in Belgium in the early 1990s (*19*), then New Zealand since 1991 (*14*). In New Zealand an epidemic with incidences up to 17.4 cases/100,000 population in 2001 prompted an immunization campaign with custom made outer-membrane-vesicle vaccine MeNZB (Novartis Vaccines and Diagnostics, Siena, Italy) (*20*).

By using cluster detection software SaTScan (www.satscan.org) (*21*) for laboratory surveillance of IMD at the German Reference Center for Meningococci (*22*), we showed spatial concentrations of meningococci with antigen sequence type B:P1.7–2.4:F1–5 (serogroup B, PorA VR1 7–2, PorA VR2 4, and FetA VR 1–5), strongly associated with cc41/44 (*11*), around the German city of Aachen near its border with the Netherlands. The annual incidence rate rose to 3.1/100,000 in 2005 among a population of 1.1 million living in Aachen and 3 neighboring counties (Greater Aachen; Figure 1).

**Figure 1:**
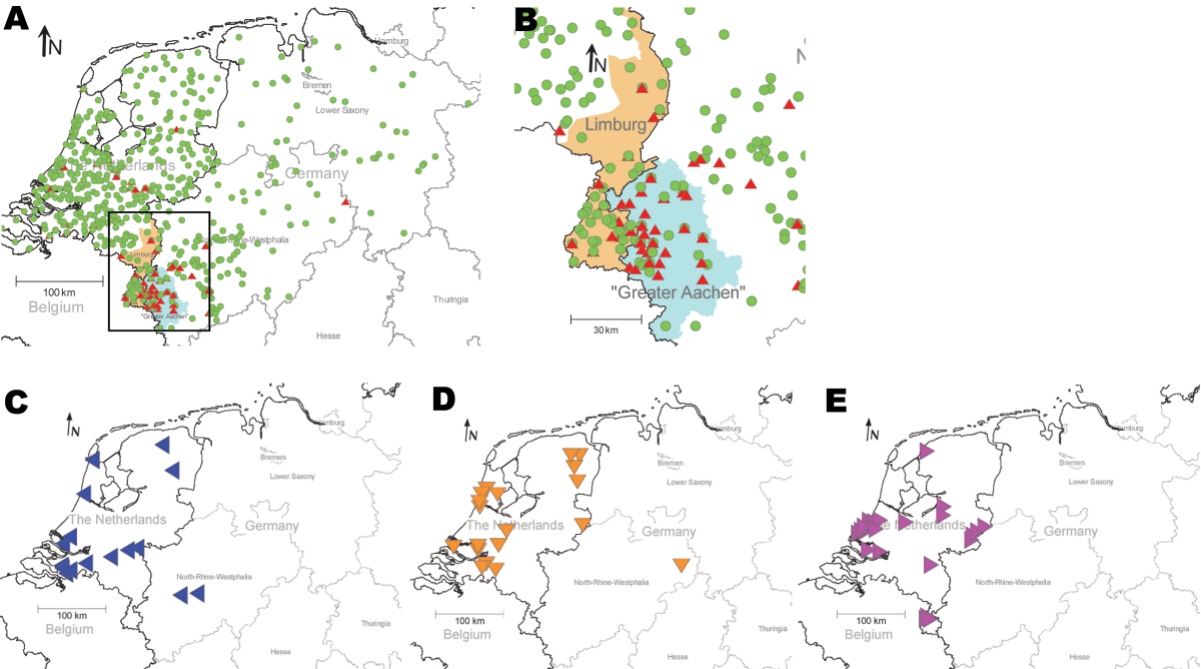
Distribution of clonal complex 41/44 *Neisseria meningitidis* strains in Germany and the Netherlands during 2001–2006 with positively associated multiple-locus variable-number tandem repeat analysis (MLVA) and multilocus sequence types (MLST). A) Distribution of MLVA type (MT) 19/MLST (ST) 42 strains (red triangles). Full green circles represent non–MT19/ST42 strains. Black rectangle delineates the area magnified in panel B. B) Area encompassing Limburg (orange shading) and Greater Aachen (blue shading). C–E) Spatial distribution of other overrepresented MT/ST variants: MT27/ST40 (blue triangles); MT30/ST40 (orange triangles); MT78/ST1374 (purple triangles).

We mapped the epidemiology of IMD caused by cc41/44 meningococci in the Netherlands and 2 neighboring German states, Lower Saxony and North-Rhine-Westphalia, during 2001–2006. Furthermore, we characterized the clone responsible for the upsurge of IMD in Greater Aachen.

## Materials and Methods

### Bacterial Strains

All *N. meningitidis* strains analyzed in this study were isolated from patients with IMD. One isolate was included for each patient. During 2001–2006, a total of 239 strains collected by NRZM and 904 isolates collected by the Netherlands Reference Laboratory for Bacterial Meningitis, Academic Medical Center, Amsterdam, North Holland, the Netherlands) were included in the study. Only strains of serogroup B with positive amplification of cc41/44-specific restriction-modification system *Neisseria meningitis* (*Nme)*SI (*17*) and subsequent confirmation of cc41/44 by MLST were included. In addition, 51 serogroup B, *Nme*SI–positive strains obtained from the Netherlands in 1985 were analyzed as a historic reference. NZ98/254 is the meningococcal strain used to make MeNZB (Novartis Vaccines and Diagnostics) (*20*).

### Typing of Meningococci

MLST (*7*) and antigen sequence typing of PorA (*23*) and FetA (*24*) were performed according to published protocols. MLVA targeting 8 loci was performed according to the method described by Schouls et al. (*10*). However, nomenclature of repeat profiles has been changed since their original description; current MLVA types (MTs) and conversion tables are available from www.mlva.net. Unique combinations of serogroup, PorA variable region 1 (VR1), PorA variable region 2 (VR2), and variable region of FetA (FetA VR) were termed fine types. Simpson diversity indices (DIs) of the above typing schemes used alone or in combination were calculated as outlined by Hunter and Gaston (*25*). Ninety-five percent confidence intervals (CIs) of DI were determined by using the percentile bootstrap method after 1,000 replicates implemented in the package boot, written by A. Canty and B. Ripley for R (R Foundation for Statistical Computing, Vienna, Austria), version 2.8.0 (www.r-project.org).

### Spatiotemporal Data

**Figure 2:**
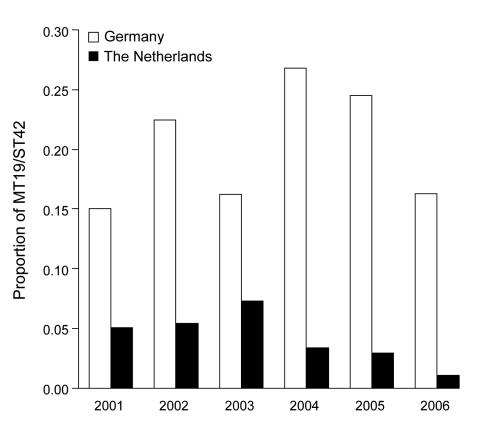
Temporal progression of the proportion of MT19/ST42 meningococcal strains in the Netherlands and the German study region (North-Rhine-Westphalia and Lower Saxony), 2001–2006.

Geographic coordinates (map datum World Geodetic System [WGS] 84) were derived from German and Dutch postal codes. Yearwise categorization of data was based on the dates of sampling or dates of entry if sampling information was not available. Maps (Figure 1) were generated by using Regiograph 8 (GfK GeoMarketing GmbH, Bruchsal, Germany). Yearwise spatial densities of MT19/ST42 strains in the study area (Figure 2) were calculated by using Spatstat, version 1.15–1, created by A. Baddeley and R. Turner for the statistical environment R.

### Statistical Analyses

Covariation among MTs and STs was assessed by using the Jaccard similarity coefficient (J), as described by Rhee et al. (*26*), with slight modifications. Briefly, for any combination of MT and ST, the J coefficient is the ratio between the number of strains belonging to the combination in question divided by the number of strains sharing either MT or ST. It is calculated as J = N_VS_/(N_VS_ + N_V0_ + N_0S_), where N_VS_ represents the number of strains with a certain MT and ST, N_V0_ is the number of strains with the same MT but another ST, and N_0S_ is the number of strains with the same ST but another MT. Observed J and expected Jaccard coefficients (J_EXP_) were compared for each combination, assuming random coupling of types. J_EXP_ was calculated as the mean J coefficient after 2,000 random rearrangements of the MT and ST vector (consisting of Boolean values indicating presence or absence of types). p values testing the equality of J and J_EXP_ for any combination of MT and ST were derived by using an inverse quantile function based on the distribution of 10^5^ bootstrap replicates. We used the Holm method to control the familywise error rate for multiple hypothesis testing (*27*). The Pearson χ^2^ test was calculated with R.

### Serum Bactericidal Assay

German strain DE9686 (B:P1.7–2,4:F1–5:ST42:MT19) isolated in 2004 from a patient of IMD in Aachen was used as the target strain in a validated serum bactericidal assay (*28*). Because the serum complement source used in the New Zealand trials contained interfering antibodies against strain DE9686, an alternative serum complement source for strain DE9686 was found from a range of adult volunteers. Prevaccination and postvaccination serum samples from 20 persons, 18 months–12 years of age, who had been vaccinated with 3 doses of MeNZB vaccine (Novartis Vaccines and Diagnostics) during the New Zealand trials, were tested against the German target strain, DE9686 by using the new serum complement source. Interpolated titer values were measured by using a formula that calculates the level of antibodies on the basis of percentage kill immediately on either side of the 50% cutoff (*28*).

## Results

### Performance of Typing Methods

All 1,143 strains were tested by MLVA, MLST, and antigen sequence typing. Fine typing, i.e., serogroup, PorA VR1, PorA VR2, and FetA VR type showed 195 unique types, which translates to a low DI of 0.752 (95% CI 0.726–0.778). MLVA and MLST distinguished 232 (DI 0.942, 95% CI 0.933–0.948) and 222 (DI 0.893, 95% CI 0.879–0.905) types, respectively, confirming the higher discriminatory ability of MLVA when compared with MLST. Both neutral typing methods (MLST and MLVA) provided higher discrimination than antigen sequence typing. Finally, combination of MLVA and MLST yielded 504 unique MLVA-MLST (MT-ST) types, demonstrating the extremely fine-grained resolution (Simpson’s index 0.985, 95% CI 0.981–0.987) attained for the binational collection of cc41/44 *N. meningitidis* strains.

### Covariation and Spatial Pattern of MLVA-MLST Combinations

Covariation was computed for MTs and STs that were identified at least 10 times. The average J coefficient for observed combinations was low (0.06), suggesting a limited overlap between MLVA and MLST. After controlling the familywise error rate at <0.01, four MT-ST combinations showed marked positive association, indicating recent clonal expansion: MT19/ST42, MT27/ST40, MT30/ST40, and MT78/ST1374. In contrast, 3 combinations occurred significantly less frequently than expected: MT27/ST41, MT30/ST41, and MT78/ST41 (Table 1). Geographic coordinates were available for 1,102 (96.4%) of 1,143 strains. Two of the positively linked MT-ST combinations showed evidence for clustering: MT78/ST1374 around the Dutch city of Den Haag and, more explicitly, MT19/ST42 in Greater Aachen (Figure 1, panels A and B).

**Table 1:** Positively and negatively correlating MLVA-MLST pairs, *Neisseria meningitis* clone, Greater Aachen Region, Germany, 2001–2006*

Association	MT	ST	N	N_EXP_	N (MT]	N (ST]	J	J_EXP_	p value
Positive	19	42	91	38	209	209	0.2783	0.1013	0
	30	40	19	3	38	82	0.1881	0.0235	0
	78	1374	25	2	54	39	0.3676	0.0199	0
	27	40	18	4	59	82	0.1452	0.032	0.00002
Negative	27	41	3	16	60	292	0.0086	0.0462	0
	30	42	1	7	38	209	0.0041	0.0287	0
	78	41	4	14	54	292	0.0117	0.0416	0.00004

*MLVA, multilocus variable tandem repeat analysis; MLST, multilocus sequent type; MT, MLVA type; ST, multi-locus-sequence type, N, no. strains with MT and ST; N_EXP_, expected number of strains with MT and ST; N (MT], no.strains with MT; N (ST], no. strains with ST; J, Jaccard index; J_EXP_, expected Jaccard index; p, 2-tailed p values (see text).

### Clustering of MT19/ST42 Meningococci

Strains with MT19/ST42 occurred almost exclusively on the German side of Greater Aachen: of 50 German MT19/ST42 strains, only 8 were isolated from outside this region, all but 1 occurred within 100 km of Aachen (Figure 1, panel B, p<2.2 × 10^–16^, χ^2^ test). A similar, albeit less marked concentration, was observed for the Netherlands regarding the province of Limburg: 15 of 37 MT19/ST42 (40.1%) strains with corresponding regional data originated from this province compared with 97 (11.7%) of 826 other cc41/44 isolates (p = 1.2 × 10^–6^, χ^2^ test). From a total of 91 MT19/ST42 strains, 81 (89.0%) were B:P1.7–2.4:F1–5. In contrast to the 41 Dutch MT19/ST42 isolates, which displayed 7 different fine types with 76% dominance of B:P1.7–2.4:F1–5, all 50 German MT19/ST42 isolates were B:P1.7–2,4:F1–5. Mean annual incidence rates of MT19/ST42 per 100,000 population in the Netherlands, the German states Lower Saxony and North-Rhine Westpahlia (including Greater Aachen), and Greater Aachen were 0.04, 0.03, and 0.63, respectively. In conclusion, most of type MT19/ST42 strains were isolated from Germany (50/91 strains), where they displayed a higher degree of clustering and antigenic uniformity.

### Temporal Trends and Migration of the Outbreak Strain

**Figure 3:**
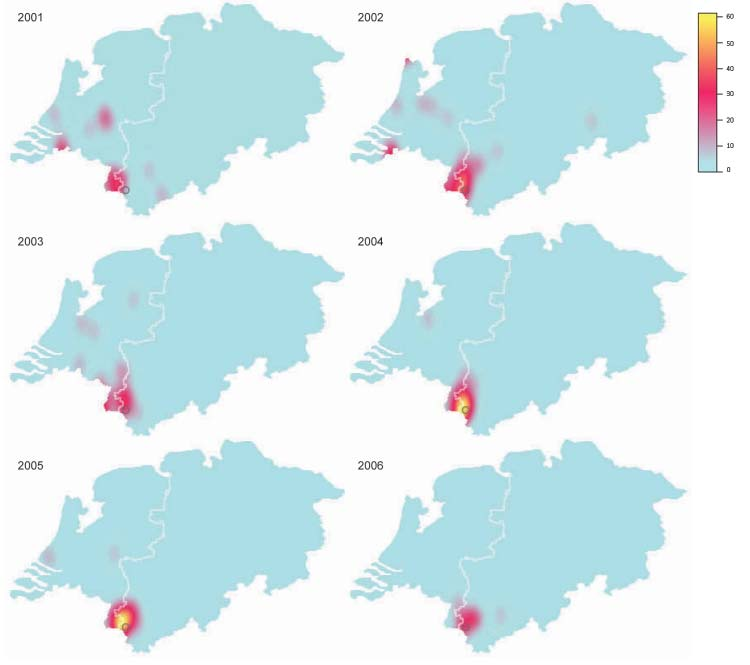
Yearwise spatial distribution of MT19/ST42 strains within the study region in Germany during 2001–2006. Color-coded values represent estimates of the intensity function underlying the point pattern data. Gray circle marks the city of Aachen; the white line represents the border between the Netherlands and Germany.

The total number of cc41/44 isolates declined from 257 in 2001 to 137 in 2006. The proportion of MT19/ST42 strains was higher in the German region in every year from 2001 to 2006 and peaked at 0.27 in 2004 (Figure 2). This clone was the most common MT-ST combination in the German region during 2001–2006, as opposed to the Netherlands, where the most numerous combinations per year were MT19/ST41 (2001), MT19/ST42 (2002), MT19/ST41 (2003), MT18/ST41 (2004), MT18/ST41 (2005), and MT19/ST41 together with MT18/ST41 (2006). Furthermore, the proportion of MT19/ST42 in the German area never fell below 0.15, whereas in the Netherlands it decreased to 0.01 in 2006 (Figure 3) because of slow eastward migration from the Dutch province of Limburg toward Greater Aachen (Figure 3). A historic sample from the Netherlands from 1985 yielded 1 MT19/ST42 strain out of 51 (proportion 1.96%, 95% CI 0.00–11.79), indicating either reoccurrence of this type over >2 decades or independent reassociation of alleles.

### Age Distribution of Case-Patients

**Figure 4:**
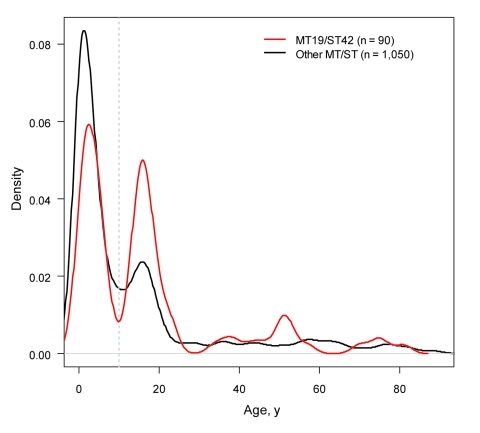
Kernel density plots of age distribution of MT19/ST42 case-patients compared with the rest of the ST41/44 complex. The vertical gray line indicates 10 years.

Age information was available for 1,140 of 1,143 (99.7%) case-patients. IMD caused by MT19/ST42 occurred more commonly among patients >10 years of age (p = 4.2 × 10^–4^, χ^2^ test). A plot of the age distribution of cases due to meningococci of cc41/44 illustrates a bimodal pattern considered typical for IMD (Figure 4). Nevertheless, cases caused by clone MT19/ST42 disproportionately affected adolescents, with no difference between the Netherlands and Germany (p = 0.90, χ^2^). This positive shift is consistent with an epidemic age pattern described in the 1970s during an epidemic wave of meningococcal disease in Finland (*29*) and during the 1980s in the Netherlands (*30*).

### Serum Bactericidal Antibody Responses against DE9686

 Typing methods (MLVA, MLST, “fine typing”) could not distinguish NZ98/254 from DE9686 (B:P1.7–2.4:F1–5:ST42:MT19). Serum bactericidal antibody responses of persons vaccinated with MeNZB suggested protective levels (i.e., >8) in all serum samples. These samples were tested in a serum bactericidal assay with DE9686 as a target strain. The test determines the maximal dilution at which killing activity of tested serum can be observed (Table 2).

**Table 2:** Serum bactericidal antibody titers before and after vaccination with MeNZB against German strain DE9686, Greater Aachen Region, Germany, 2001–2006*

Age group	No. vaccinees	Before	After	4-fold rise	>8 post
8–12 y	1	12	1,727	←	←
	2	3	104	←	←
	3	189	406	–	←
	4	50	296	←	←
	5	229	729	–	←
	6	23	175	←	←
	7	9	84	←	←
	8	2	213	←	←
	9	2	77	←	←
	10	2	55	←	←
18–24 mo	11	2	214	←	←
	12	4	45	←	←
	13	2	109	←	←
	14	7	92	←	←
	15	2	306	←	←
	16	6	431	←	←
	17	2	216	←	←
	18	3	44	←	←
	19	3	218	←	←
	20	2	171	←	←

*– represents cases where conditions 4-fold rise and >8 post are false. Arrows represent cases where conditions 4-fold rise and >8 post are true.

A 4-fold rise in titer was observed in the postvaccination sample for all (10/10) toddlers (18–24 months of age) and 8 of 10 schoolchildren (8–12 years of age). Only titers in 2 persons with the highest prevaccination titers (189 and 229) rose <4-fold after vaccination with MeNZB (Novartis Vaccines and Diagnostics).

## Discussion

Our main goals were to identify the clone causing the rise in incidence rate in Greater Aachen and to elucidate whether increased disease activity in Germany represented local emergence or cross-border spread from the Netherlands, which had experienced a steep rise in IMD caused by cc41/44 since 1980 (*18*). Tracking of variants within cc41/44 necessitated a high level of discrimination, achieved by the combination of typing methods MLVA and MLST (DI 0.985). Geographic mapping showed it was only the latter pairing of techniques that sharply delineated a spatial accumulation of MT19/ST42 meningococci in the region that had seen increase of disease rate (Figure 1). The spread of the outbreak clone differed upon introduction into Germany, where it caused high levels of disease in a confined area. The distinct level and high spread of disease may suggest the presence of regionally specific, as yet unknown, factors contributing to its emergence. Because behavior-related risk factors promote acquisition of IMD in adolescents (*31*), locally differing traditions in Germany, possibly related to carnival festivities in seasons with high incidence (*32*), may have contributed to the outbreak. Nevertheless, clustering was also present, albeit less abundantly, in the Dutch province of Limburg. Higher antigenic diversity and discrete eastward motion indicate the clone’s longer history in the Netherlands, where it had failed to cause an epidemic, possibly due to population immunity elicited by long-lasting exposure to related variants of cc41/44 since the 1980s.

Although clusters of cases tend to be short-lived in industrialized countries (*33*), the geographic concentration of the outbreak clone was observed during the whole study period (Figure 3). This spatial stability might be promoted by the clone’s more efficient evasion of induction of mucosal immunity. Notably, antigenic variation of the outbreak clone was limited and dominated by B:P1.7–2.4:F1–5 (89%). Lower antibody avidity observed after vaccination with OMVs containing P1.7–2.4 (*34*) suggests that this PorA-type evokes a less potent immune response, possibly leading to decreased protection against acquisition of carriage. Studies confirming this hypothesis, however, have not been published. Moreover, the higher diversity unveiled by neutral typing techniques compared with antigen sequence typing could suggest positive selection for strains achieving immune escape because of their antigenic profile.

There was a significant shift toward older age of patients infected by MT19/ST42 meningococci, consistent with observations before and during epidemics (*29**,**30*). A recent report demonstrates an overrepresentation of meningococci harboring the meningococcal disease associated island among young adults with IMD, possibly indicating its contribution to invasive disease in this age group (*35*). Frequent isolation of presumably more virulent meningococci, such as MT19/ST42, from adolescents might be explained by the hypotheses that 1) fewer virulence determinants are required to cause invasion in infants, hence strains of lower invasiveness are recovered in higher proportions among them, and 2) invasiveness represents a smaller penalty for highly transmissible strains in persons with abundant social contacts (*36*), leading to their preferential circulation among older age groups.

In an analysis of meningococci across several clonal complexes, Schouls et al. obtained similar groupings by MLVA and MLST (*10*). To identify type pairs deviating from their expected occurrence within strains of this study, which pertained to a single clonal complex (cc41/44), we computed the degrees of overlap (represented by J coefficients) of observed combinations between MTs and STs. The mean overlap was low (0.06), and the number of most combinations did not differ significantly from the expected, suggesting random association in most types and highlighting the complementary nature of MLST and MLVA for cc41/44. The added value of combining these typing methods is also reflected in the significantly higher DI attained. Positively associated combinations could reflect linkage disequilibrium in concordance with an epidemic population concept (*37*), which attributes disequilibrium to transient multiplication of successful variants doomed to dissipate within years, secondary to recombination. Nevertheless, recovery of MT19/ST42 over >20 years favors concepts that accommodate the observed excessive stability, e.g., proposed in models including interstrain competition (*36*). On the other hand, strains with a negative association might indicate low epidemic potential. All sequence types recovered from covariation analysis (ST40, ST42, ST41, and ST1374) represent (sub)group founders within cc41/44, attributed higher transmissibility and fitness due to persistent recovery in both carrier and invasive collections (*36*). Although observed presence of founder STs could be due to biased selection of STs for covariation analysis (only types occurring at least 10 times were included), the clear underrepresentation of some MTs belonging to mentioned STs suggests that at least virulence within these founder STs is not equally distributed (Table 1).

The following observations support the hypothesis that meningococci of the clone MT19/ST42 command exceptional epidemic potential: dramatic spatial concentration displayed in Greater Aachen, concurrent rise of incidence in the area of clustering, and age-shift to older patients. Tendency to affect older persons has also been noted during epidemics (*29*,*30*) and in strains carrying a temperate bacteriophage associated with higher pathogenic potential (*35*). In addition, vaccine strain NZ98/254, which was used for generation of New Zealand’s MeNZB (Novartis Vaccines and Diagnostics) (*38*), was not distinguishable from German MT19/ST42 strains, demonstrating the clone´s emergence on separate continents.

By using paired serum samples from vaccinated toddlers and schoolchildren (Table 2), we were able to show a striking similarity between the serum bactericidal antibody responses induced by the German epidemic strain and results obtained in New Zealand against NZ98/254 after vaccination of toddlers and 8–12 years of age (*39**,**40*). Similar to the New Zealand situation, a protective vaccine effect was strongly supported by the serum bactericidal antibody responses induced by the German strain. Moreover, because the VR2 epitope of PorA is the major target for immune response elicited by MenZB (*38*), protection against most of the analyzed cc41/44 strains (58% have VR2 type 4) can be assumed.

Although the marked concentration of meningococci with fine type B:P1.7–2.4:F1–5 continues to exist in western North-Rhine-Westphalia (www.episcangis.org), incidence rates have been decreasing since 2005; this decrease has suspended plans to implement a vaccine campaign. Nevertheless, the New Zealand experience (*14*) suggests that this clone may attain high-level endemicity, and continued close surveillance remains a task of high priority. Should regional incidences rise again, implementation of an immunization campaign with MeNZB should be considered.

Although we studied a large area involving 2 bordering countries, our study is limited. We did not include isolates from asymptomatic carriers because of the logistic difficulties related to the collection of representative carriage samples that preceded or temporally coincided with outbreaks. Furthermore, the sampling period only covered 6 years with 1 historic reference year because of the large number of strains. Finally, representative population immunity data were not available. Our study does, however, pave the ground for future epidemiologic and experimental work aimed at confirming the distinct pathogenic potential of MT19/ST42 meningococci and unraveling the circumstances leading to their spatially distinct occurrence.

We tracked an outbreak clone that was causing considerable disease activity on the border of 2 industrialized European countries by using highly discriminatory and portable typing techniques. These techniques could guide and improve the targeting of public health efforts, which may include vaccination, if incidence rates in North-Rhine-Westphalia begin to rise again.
